# Tools for ligand validation in *Coot*

**DOI:** 10.1107/S2059798317003382

**Published:** 2017-03-06

**Authors:** Paul Emsley

**Affiliations:** a MRC Laboratory of Molecular Biology, Francis Crick Avenue, Cambridge Biomedical Campus, Cambridge CB2 0QH, England

**Keywords:** *Coot*, ligand validation, model building, ligand representation, ligand manipulation

## Abstract

*Coot* is a molecular-graphics program primarily aimed at model building using X-ray data. Recently, tools for the manipulation and representation of ligands have been introduced. Here, these new tools for ligand validation and comparison are described. Ligands in the wwPDB have been scored by density-fit, distortion and atom-clash metrics. The distributions of these scores can be used to assess the relative merits of the particular ligand in the protein–ligand complex of interest by means of ‘sliders’ akin to those now available for each accession code on the wwPDB websites.

## Abstract 2

The current tools of *Coot* for ligand validation and comparison in are presented. The user-selected ligand is assessed by ligand-distortion and map-correlation metrics, and compared with those of ligands of the wwPDB to create a percentile rank.

## 1. Introduction   

For many years, the validation of macromolecular structures has been a concern of practicing crystallographers and users of the PDB (Berman *et al.*, 2000 ▸) (and more recently the wwPDB; Berman *et al.*, 2003 ▸); see, for example, Brändén & Jones (1990 ▸), Dodson (1998 ▸) and Davis *et al.* (2007 ▸). Since 2007, crystallographic diffraction data deposition has been mandatory for structure depositions at the wwPDB sites. This, and the increase in the number of deposited structures, has enabled macromolecular model validation to be reconsidered (Read *et al.*, 2011 ▸) and the recommendations that were made have been implemented by the wwPDB deposition sites to provide access to a concise summary of well established quality indicators.

Using these global metrics of structure quality, the users of crystallographic models have been able to assess the overall quality of models. However, the assessment of the quality of local regions, and in particular ligands, has needed more consideration and effort, and the interpretation of ligand density and pathology of the atomic displacement parameters has been problematic (Lamb *et al.*, 2015 ▸).

To address this, in the context of information presented about ligands by wwPDB sites, validation of ligands and protein–ligand complexes has been proposed (Adams *et al.*, 2016 ▸). Building upon the *Coot* ligand tools described previously (Debreczeni & Emsley, 2012 ▸), the tools and valid­ation described here bear some relation to the recommendations therein.

A number of publications and services that have been, to varying degrees, inspiration for the current work will be discussed briefly. Weichenberger *et al.* (2013 ▸) noted that the real-space correlation coefficient (RSCC) provides a good measure of the fit of residues (and ligands) to the electron density. The *Twilight* web server provides a spreadsheet of ligands from the wwPDB that have had their RSCC assessed.

The Validator^DB^ web site (Sehnal *et al.*, 2015 ▸) available at http://ncbr.muni.cz/ValidatorDB offers additional ligand valid­ation of deposited structures, with a particular focus on chirality.

*Mogul* (Bruno *et al.*, 2004 ▸) is software available from the Cambridge Crystallographic Data Centre (CCDC) that uses a knowledge base derived from the Cambridge Structural Database (CSD) to provide information on preferred bond lengths, angles and other geometric criteria. The input is a query in the form of a bond or angle description (or, more generally and typically, a number of these derived from a molecular description provided in the form of a PDB file or an MDL MOL file). *Mogul* has been used to assess the torsion strain energy of ligands in the PDB (Liebeschuetz *et al.*, 2012 ▸); the authors focused on torsions as bond and angle values are more influenced by refinement target values.

*VHELIBS* (Cereto-Massagué *et al.*, 2013 ▸) is a user interface that helps in the validation of ligand-binding sites by allowing the user to visually assess the fit of the ligand to the map, and assesses ligands and the surrounding residues as ‘Good’, ‘Dubious’ or ‘Bad’.

## 2. Methods   

The ligand-selection and scoring methods are described below.

### 2.1. Choice of ligand   

It is not always clear from the content which ligand in a protein–ligand complex was of interest to the original authors. The ligands in this analysis were selected as follows.

For a given coordinate file the nonpolymer residue types are enumerated. The largest ligand (judged as that with the most non-H atoms for the given residue type in the dictionary) is selected for analysis. There are a number of criteria to pass before further analysis.

(i) The residue type is not annotated as obsolete in the Chemical Component Dictionary (CCD; Westbrook *et al.*, 2015 ▸) entry.
(ii) The selected residue has no associated LINK record.
(iii) The selected residue has no atoms in alternate configurations.
(iv) The data are available and are not twinned.

### 2.2. Choice of metrics   

Following similar reasoning to that of Weichenberger *et al.* (2013 ▸), the RSCC of the ligand-omitted 2*mF*_o_ − *DF*_c_ map (here called the ‘direct map’) at the ligand site was chosen as one of the metrics.

The selected ligand is removed from the set of atoms from which structure factors are calculated but, using appropriate *REFMAC* (Murshudov *et al.*, 2011 ▸) keywords, the ligand coordinates are used for mask calculation.

One would imagine that in a well refined model there would be little to no residual difference map density at the site of the ligand. The RSCC of the difference map (as output by *REFMAC*) is also considered (in this case, the ligand is not omitted from the structure-factor calculation). Density-grid coordinates that have density contributions from neighbouring residues are masked out. The atomic radii used for masking follow a similar function to that used in *REFMAC* for adding density contributions.

The current set of ligand-validation tools described here does not include chiral centre validation. It is straightforward to check ligand models against the chiral centre definitions in the *REFMAC* monomer library (where chirality is described locally), but it is technically challenging to be able to convert neutral-ligand *R*/*S* Cahn–Ingold–Prelog (CIP) chirality as described in the CCD to a form that is useful for comparison (for example, the deprotonation of phosphates can lead to the removal of chiral centres).

Additionally, the validation of chiral centres produces a result that is yes/no, which is a different form to the sliding-scale results produced by the other validation tests described here (which means that sliders would not be the most useful representation of chiral validation information). Refinement with inverted chiral restraints leads to distortions in bonds and angles, and these features can be detected by the current metrics.

### 2.3. Difference-map analysis   

Tickle (2012 ▸) noted that there are problems with using the real-space correlation and real-space *R* factor because the values reflect both accuracy and precision. An alternative electron-density validation statistic using the difference map is proposed, with the challenge being to formulate an effective metric. Tickle promotes the use of *Q*–*Q* (quantile–quantile) plots for Δρ, and these are now available in *Coot*.

Differences from the diagonal line indicate that the observed difference map does not conform to that expected from a normal distribution of errors. The metric in *Coot* is not global, since it only measures the distribution of the difference map in the context of the specified residue (typically the ligand) and its environment. In due course, analysis of the density in the solvent region (which would largely be un­influenced by errors in the atomic model) would provide an estimate of σ(Δρ) and hence *Z*_Δρ_, a measure of ligand model accuracy (see §5.2 of Tickle, 2012 ▸).

### 2.4. Effective resolution   

Using the nominal resolution (the *R*_nom_ presented in the data file) to describe the quality of a data set can be misleading (for example in the case of unusually weak or incomplete data; Weiss, 2001 ▸). Measures to address this shortcoming have been published (Urzhumtseva & Urzhumtsev, 2015 ▸). Here, a modified value, *R*_eff_, was used, taking into account missing data and the standard deviations of the reflection amplitudes (Murshudov, 2016 ▸). See Appendix *A* [App appa] for for the derivation of *R*_eff_.

### 2.5. Bad contacts   

H atoms are added to the model ligand and its environment using *Reduce* (Word, Lovell, Richardson *et al.*, 1999 ▸). Information about the ligand bonding (including bonding information about the H atoms) is generated from the mmCIF ligand dictionary and written to a connections file for use by *Reduce*.

*Probe* (Word, Lovell, LaBean *et al.*, 1999 ▸) is used to generate atom contacts between the ligand and its environment. The number of atom pairs that have bad overlaps between them are recorded for each ligand. In future an overlap volume will be used, which will hopefully be more precise than the integers that this module currently generates.

### 2.6. *Mogul* *Z*-worst   

The canonical source of molecular information for this ligand analysis is the entry for the ligand in the CCD (the term ‘comp-id’ will be used to represent the three-character alphanumeric code that the wwPDB assigns to each chemical component). The atom and bond information is extracted and is combined with the coordinates of the atoms for the selected ligand in the model to construct an internal representation of the molecule (Landrum, 2010 ▸). Not all molecules pass this step. An example of a failure is the ligand with comp-id 1MK, where the molecular-sanitization (a check that the valence, aromaticity, hybridization and conjugation of the molecule are consistent) step fails.

Using *RDKit* and the *RDKit* molecule-export function, an MDL MOL file (MDL Information Systems Inc., San Leandro, California, USA) is generated as an input query for *Mogul*. Some functional groups are modified as needed to comply with the query preparation (*Mogul* User Guide and Tutorials, §2.3).

A simple *Mogul* control script is generated corresponding to the MDL MOL file, and the *Mogul* executable is invoked as a separate synchronous process. Upon termination, the *Mogul* output file is parsed, converting atom indices back to atom names, allowing the representation of model geometrical parameters in relation to the distribution of preferred values corresponding to the relevant crystal structures in the database.

The geometric parameter is compared with the mean and standard deviation of the preferred values and a *Z*-value is generated for that geometric parameter. This is repeated for all bonds and angles in the ligand. Thus, we have a number of *Z*-values: that chosen to represent the molecular geometry is ‘*Mogul* *Z*-worse’, the geometric parameter that has the highest absolute *Z*-value.

For some unusual bonds or angles *Mogul* will only have a handful of structural representatives (perhaps correlated), the distribution of which will have unusually small standard deviations. Making strong claims about problematic geometry for which we have little prior knowledge should be avoided in such cases, thus a lower-bounds σ-cutoff is introduced (0.015 Å for bonds and 1° for angles). This will consequently lower the resulting *Z*-value for the given geometric feature.

### 2.7. *EDSTATS*   

*EDSTATS* (Tickle, 2012 ▸) was run for all of the ligands and the statistics for the ligands were collected. The statistics from *EDSTATS* were not used directly as part of the scoring system but are available for exploitation by others.

### 2.8. Combining ranks   

Once we have percentiles/ranks for individual metrics, we can rather straightforwardly combine these ranks to obtain an overall score, *S*, which can then be used itself to rank the ligands. There are a number of plausible ways to combine individual scores; that chosen currently (1) spreads out the top end of the scores compared with linear addition. A future implementation may use non-unit weights to allow for the fact that the ligand density correlation is probably more important than the others.
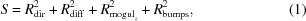
where *R*_dir_ is the rank of the direct-map correlation, *R*_diff_ is the rank of the difference-map correlation, 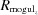 is the rank of the *Mogul Z*-worst score and *R*_bumps_ is the rank of the ligand bad-contacts score.

The values of *S* are typically then ranked and expressed as an overall percentile.

### 2.9. Implementation   

All ligand metrics have been entered into an SQLite database generated from text parsing of the log or other output files of the various programs used. The program to compile the database is part of the *Coot* distribution and is called *coot-make-ligands-database*. An additional stand-alone program *(coot-ligand-percentiles*) is available that provides indices into the distribution given metric scores.

The ligand-statistics generation interface is written using the correlation functions of *Coot* (which are in turn based on the map calculations of *Clipper*; Cowtan, 2003 ▸), and interfaces to *Probe* and *Reduce* from *MolProbity*, *EDSTATS* from *CCP*4 and *Mogul* from the CCDC using the scheme-based API in *Coot*. The SQLite database interface is optionally compiled, and is written, like the bulk of *Coot*, in C++. The representation of the metrics and percentile ranks is written in Python and uses GTK.

The code described here is part of the current *Coot* source-code distribution (v.0.8.7 at the time of writing).

## 3. Results   

The ligand-selection system often picks buffer molecules or cofactors. In the case of cofactors, these may not have been the main ligand of interest in the model.

The ten most common comp-ids are PG4, CIT, MPD, MES, NDP, ADP, FMN, NAP, NAD and FAD, which constitute 25% of the ligands in this analysis. It is plausible that this is not an optimal composition of ligands for use as a reference; this suggests that the ligand array might usefully be split into buffers, cofactors and ‘others’.

### 3.1. Histograms   

With the statistics collected into a database, the data can be queried to search for a number of trends: for example, is there an improvement in ligand density correlation as time progresses (*i.e.* with PDB deposition date)? Additionally, is there a date-dependent improvement in the geometry of ligand bonds and angles?

### 3.2. Resolution-dependence of metrics   

**Figure 1:**
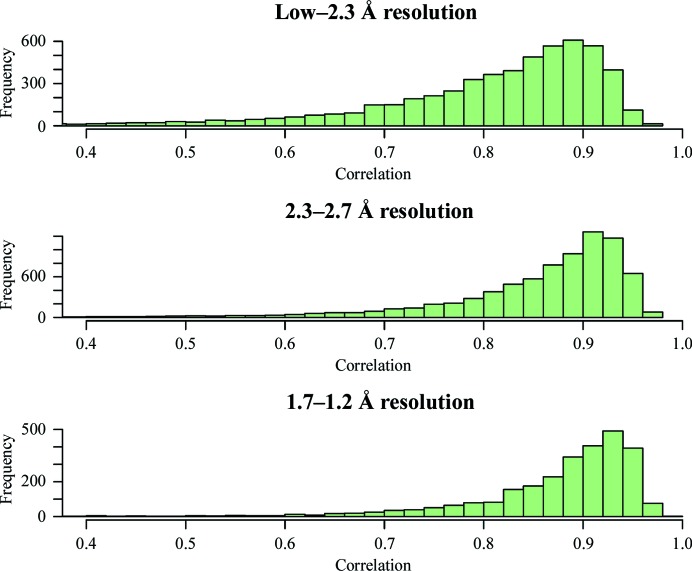
Resolution-dependence of the ligand direct-map correlations. There is a mild change in the shape of the distribution for low-resolution structures. There are relatively more structures with mediocre and poor correlations compared with the ligand correlations for medium- and high-resolution ligands. There is no resolution-dependence of the distribution of the correlation coefficient in the current model.

We can split the metrics into resolution bins to see whether there are changes in the shape of the distribution of ligand correlations and geometry distortions as a function of resolution (Figs. 1 ▸ and 2 ▸).

There is a mild shift of the distribution to lower correlations for the relatively few low-resolution ligand structures. This change in distribution as a function of resolution is not part of the model when scoring input ligands against structures in the database.

One would expect that for a well refined ligand, the density at the ligand site would be fully explained by the atomic model of the ligand (noting that regions of density with contributions from neighbouring residues are excluded from the analysis): ideally, the density at the ligand should be merely low-level noise drawn from a Gaussian distribution. However, this seems not to be the case for many ligands, in particular those that are negatively correlated with the difference map. The overall correlation to the negative difference map has a non­zero median value of −0.073 (Fig. 3 ▸). To investigate whether this negative correlation was owing to incorrect modelling of the occupancy, several data sets with negative correlation were selected and were re-refined with *REFMAC* with varying occupancies. Fig. 4 ▸ shows the variation of the correlations of the difference map and the direct map as a function of ligand occupancy. Indeed, in most cases the correlation to the difference map can be brought down to expected values, while retaining a high direct-map correlation by reduction of the ligand occupancy (typically, in these examples, to a value of between 0.6 and 0.7).

**Figure 2:**
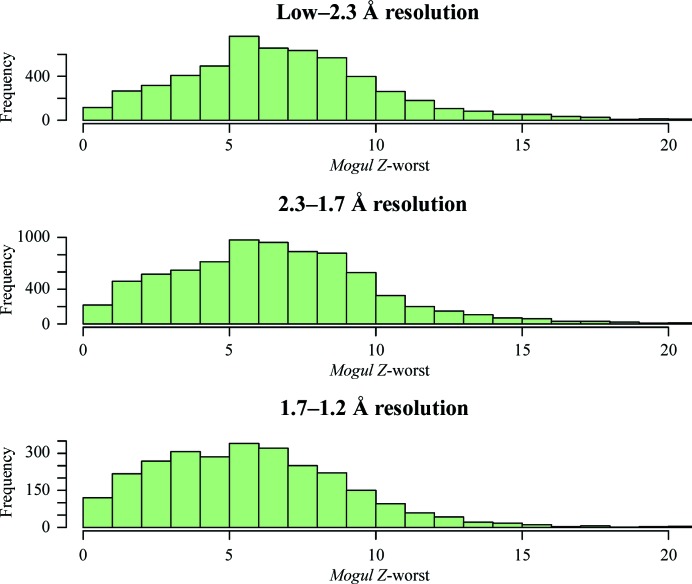
Figure illustrating the resolution-dependence of the *Mogul Z*-­worst value of the ligand. There is little to no resolution-dependence of the *Mogul Z*-­worst values.

**Figure 3:**
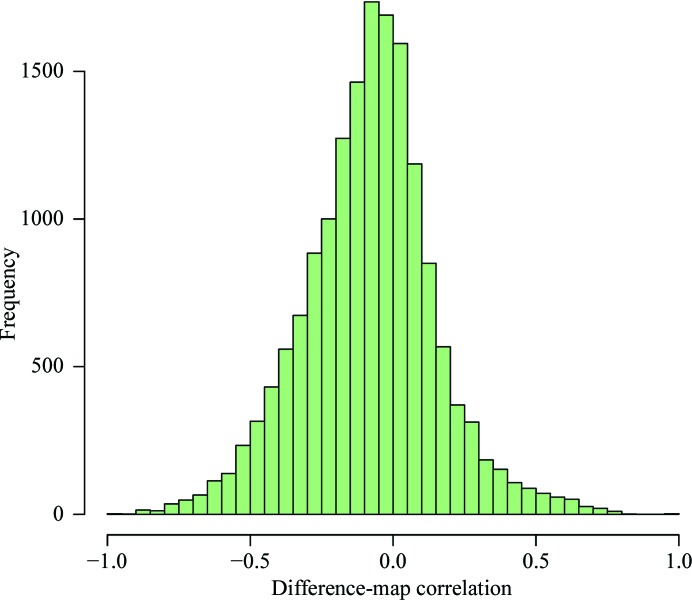
Histogram of difference-map correlations of the ligand. The histogram has a large spread and the median is slightly negative, perhaps indicating that many ligands have too much density at the ligand site, which is a result of overestimation of the occupancy.

**Figure 4:**
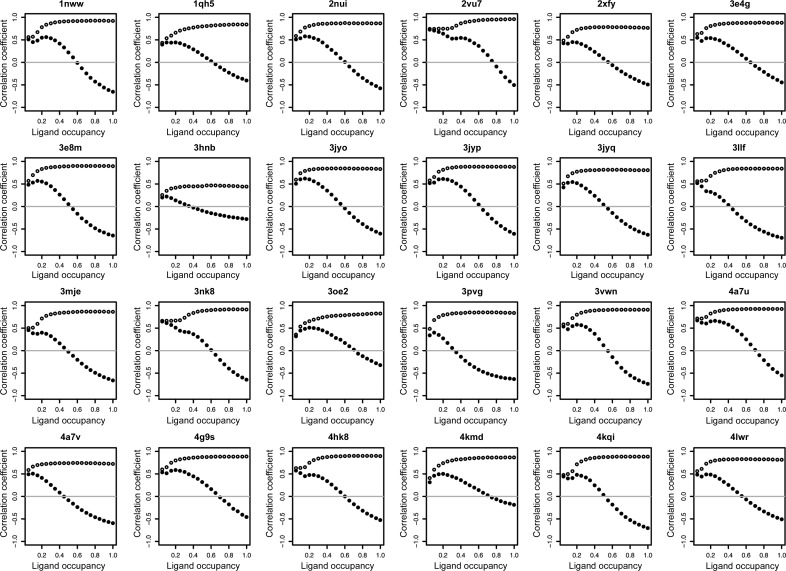
Effects on the difference-map (solid circles) and direct-map (open circles) correlations by varying the occupancy of the ligand. For each of the given structures the occupancy of the selected ligand was varied from 0.0 to 1.0 in steps of 0.05. The resulting model was refined with *REFMAC* for 30 steps. The *REFMAC* output model was tested for ligand–map correlations. The difference-map correlation can be brought down to expected levels (about 0) by decreasing the occupancy of the ligand. These structures were selected on the basis of having a combination of high-resolution data and (for the deposited structure) an unusual correlation of the ligand density and the difference map.

There is very little change in the distribution of *Mogul Z*-worst values as a function of resolution (Fig. 2 ▸). This is perhaps surprising because one might have imagined that if better (which is to say, higher resolution) data were available, it would be more easy to model the ligand with geometry values corresponding to low strain. However, this seems not to be the case. This might reflect historical refinements that were made against low-quality ligand-restraint dictionaries. It is hoped that in the future, with increasingly easy access to the sophisticated validation tools that are available (including those discussed here) for both the wwPDB deposition sites and the person solving the crystal structure in the first place, that the rate of high-quality protein–ligand complex structures will increase.

### 3.3. Sliders   

**Figure 5:**
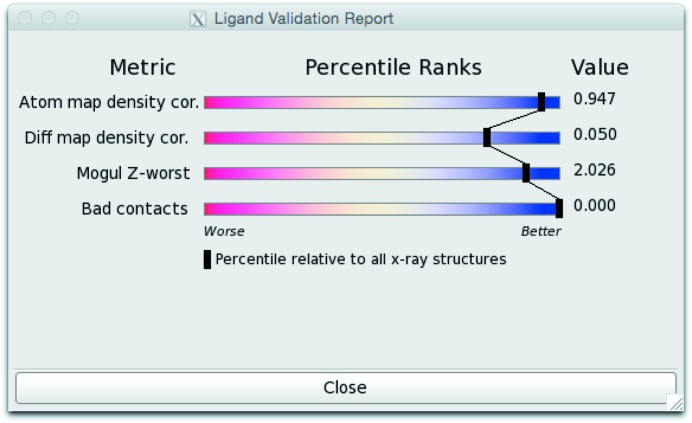
Screenshot of the sliders for a particularly well scoring ligand structure (PDB entry 4zzn).

With the distributions in place, we can compare them with the metrics generated for the particular ligand under investigation (‘how does this ligand compare to all ligands from the wwPDB?’). Radar charts have been used to represent statistics from macromolecular models (Urzhumtseva *et al.*, 2009 ▸). In this case, the percentile rank is then plotted in a similar fashion to all-molecule percentile ranks as described by Read *et al.* (2011 ▸) (Fig. 5 ▸).

### 3.4. Ligand-interaction representation   

#### 3.4.1. Three-dimensional representation   

**Figure 6:**
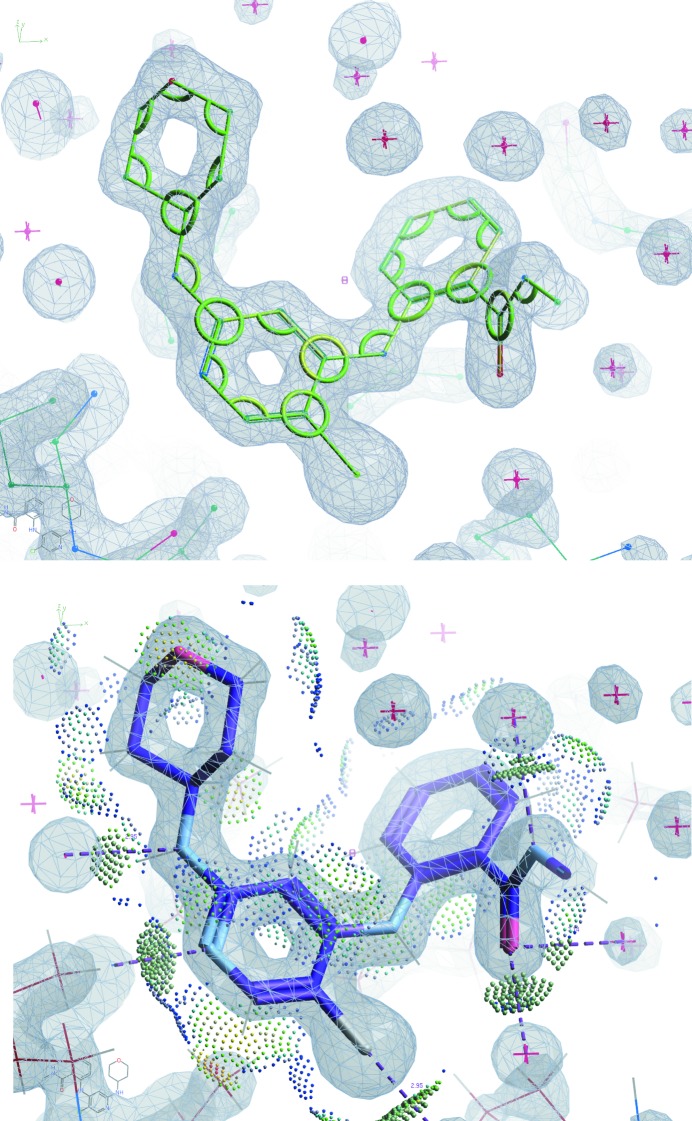
The new ligand validation in *Coot* in action. Shown here is the ligand CQ8 from PDB entry 4zzn. The ligand model corresponds well to the density map (shown in both panels) and (from the top panel) the bond and angles of the ligand model correspond well to expected values [the *Z*-values for each bond and angle are displayed on a sliding scale, with green corresponding to the most likely values (*Z*-values of 1 or less) and red corresponding to *Z*-values of 5 or greater]. The hint of red that one can see at the bottom right comes from the underlying molecule representation of ketone double-bonded oxygen. The lower panel illustrates that the manner of interaction of the ligand with the protein environment is *via* hydrogen bonds of typical length and that there are no bad contacts between the ligand and the protein. The colour scheme of the dots is as follows: hydrogen bonds are olive-green dots, wide contacts are purple, close contacts are light green, small overlaps are orange and bad contacts (not appropriate for this ligand model) would be red.

The lower panel of Fig. 6 ▸ shows the interaction of the ligand with its environment represented as coloured dots. The dots are achieved by running *Reduce* and then *Probe* in a similar fashion to that described above. In this case, however, instead of enumerating the bad contacts in the output file, the output of *Probe* is used to represent the interactions. This tool can currently be activated by the ‘Isolated Probe Dots’ menu item of the ‘ Ligands’ menu.

#### 3.4.2. *FLEV*   

**Figure 7:**
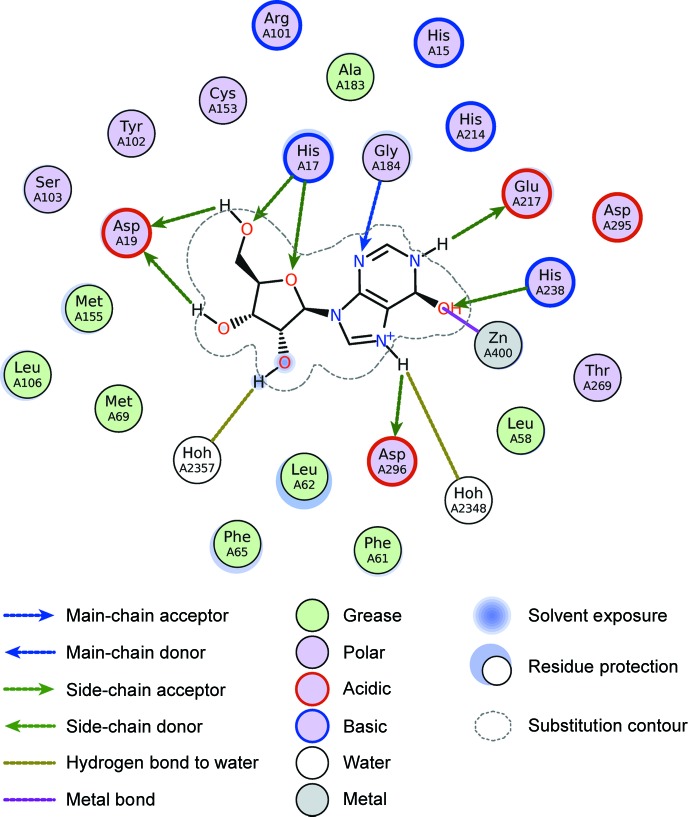
An example of *FLEV* using the 6-hydroxy-1,6-dihydropurine nucleoside in PDB entry 1a4m (Wang & Quiocho, 1998 ▸).

The mode of *Lidia* known as *Flatland Ligand Environment View* (*FLEV*) provides a means to represent the protein–’’;ligand complex in the the style of Clark & Labute (2007 ▸), which aims to provide an information-rich figure that contains important distances and interactions, and at the same time is aesthetically pleasing. The chemical diagram component of the figure is created using the Compute2DCoords() function of *RDKit*, to which a distance matrix and weight can be passed to allow, to some extent, the preservation of three-dimensional distances in the two-dimensional layout; an example is shown in Fig. 7 ▸.

## 4. Conclusions   

Ligand-analysis tools have been integrated into the new version of *Coot* and have been used to assess the ligands of structures in the PDB. The resulting metrics can be used to assess the quality of any particular ligand under refinement or due for PDB data deposition. The combined ranks score gives a single number which combines all of the metrics for a quick assessment of a particular ligand.

Analysis of the ligand metrics (Figs. 1 ▸ and 2 ▸) shows little variation as a function of resolution and thus, at least in the current implementation, the resolution of the data set is not part of the model of the expected statistics. In future, as more modern dictionary generators, refinement and model-building tools become used routinely, there may well be a substantial resolution-dependence of ligand metrics and this will need to be part of the model. This will have to be investigated in due course.

The negative median value of the difference-map correlation and the fact that this can be brought down to around zero whilst still retaining a high correction to the direct map suggest that many ligands in protein–ligand complexes are added with an occupancy of 1.0: this is too high and suggests that ligand-occupancy refinement should be a routine part of the refinement process.

**Figure 8:**
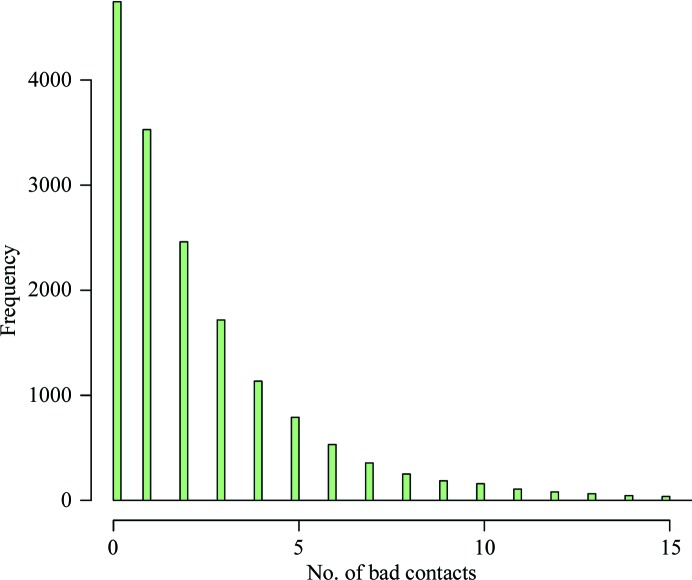
Histogram of the number of bad contacts of ligands with their environment for ligands in the wwPDB. The median number of bad contacts for a ligand is 1. Instead of aiming to be better (fewer bad contacts) than the median number of bad contacts, it is recommended to target a value of zero bad contacts.

The median number of bad contacts of PDB ligands is 1 (Fig. 8 ▸). As a guideline, it is probably a good idea to try to better this and aim for a score of 0. Using the ‘Isolated dots for this Ligand’ interface to *Probe* in the new *Coot* interface allows the straightforward determination of any problematic interactions. These can be either remodelled by adjusting the coordinates of the model or (in some cases) suggest an adjustment of the H-atom nonbonded contact interactions used during refinement.

After the per-accession-code combined scores, *S*, have been calculated and sorted, the top-ranked nonbuffer ligand is from a structure deposited by a pharmaceutical company; this is consistent with the observation of Sehnal *et al.* (2015 ▸):

the overall quality of experimental drugs is clearly much higher than the PDB-wide statistics for all ligands and non-standard residues.

It might be reasonable to change from unit weights, so that (for example) the *R*_dir_ rank is more highly weighted than the others, but this has not as yet been implemented. The top-ranking nonbuffer ligand using the current scoring system was the ligand in PDB entry 4zzn (Ward *et al.*, 2015 ▸; Fig. 6 ▸).

This analysis does not cover geometric features such as distortions from planarity of aromatic, delocalized or other *sp* ^2^-hybridized systems. To make claims about distorted plane geometry one must have a reliable understanding of chemistry, and until recently it was not clear that dictionary generators could provide this.

Although the values from wwPDB ligands are in the database, this analysis does not score (generate a percentile rank) and provide slider representation for the RSZD and RSRZO scores from *EDSTATS* for ligands to be evaluated. This should be straightforward to implement in the future.

The software on which this analysis depends is readily available (and freely available for academics) on the desktop (Mac OS X and other Unix-like systems), with the exception of *Mogul*. To increase the availability of these analyses it is hoped that in the future *AceDRG* (Long *et al.*, 2017 ▸) output will be sufficiently robust that, instead of using *Mogul*, the test of ligand internal geometry can be performed against a dictionary (quite possibly that with which the structure was refined).

This analysis does not take linked ligands into consideration. A useful addition would be to extend the set of ligands able to be assessed to include peptide ligands.

## Appendix A Effective resolution

Let us define the effective number of reflections *N*_eff_ as the sum of some function ρ which takes as parameters the observed data and the Wilson sigmas,

If all reflections in a sphere are ideally measured then the correlation between these reflections and the true reflections is 1.0 and, similarly, if these reflections have random measurement errors then the correlation will be less than 1.0. The effective number of observations can be constructed from the sum of correlations between measured reflections and true reflections. We then ask ‘what is the radius of the sphere that has this number of observations?’ Given *N* as the number of possible observations up to resolution *s* and that the number of observations is proportional to the volume of a sphere, then

and thus

so, in terms of ångströms, the effective resolution *R*_eff_ will be

Now we need to calculate ρ(*F*_o_, *F*_t_). Assuming that

where *n* is noise, assuming that the signal (*F*_t_) and noise are independent and that the average noise is 0, we obtain

〈*F*_t_^2^〉 is Wilson’s sigma and 〈*n* ^2^〉 is the variance of the noise (experimental sigma).

Thus, *R*_eff_ will have a lower (‘worse’) resolution for data that, for a given nominal resolution, have lower *F*/σ(*F*) values.
